# Histamine and histidine decarboxylase: Immunomodulatory functions and regulatory mechanisms

**DOI:** 10.1111/gtc.12774

**Published:** 2020-05-12

**Authors:** Takashi Moriguchi, Jun Takai

**Affiliations:** ^1^ Division of Medical Biochemistry Tohoku Medical and Pharmaceutical University Sendai Japan

**Keywords:** bacterial artificial chromosome, histamine, histidine decarboxylase, transgenic mouse

## Abstract

Histamine is a bioactive monoamine that is synthesized by the enzymatic activity of histidine decarboxylase (HDC) in basophils, mast cells, gastric enterochromaffin‐like (ECL) cells and histaminergic neuronal cells. Upon a series of cellular stimuli, these cells release stored histamine, which elicits allergies, inflammation, and gastric acid secretion and regulates neuronal activity. Recent studies have shown that certain other types of myeloid lineage cells also produce histamine with HDC induction under various pathogenic stimuli. Histamine has been shown to play a series of pathophysiological roles by modulating immune and inflammatory responses in a number of disease conditions, whereas the mechanistic aspects underlying induced HDC expression remain elusive. In the present review, we summarize the current understanding of the regulatory mechanism of *Hdc* gene expression and the roles played by histamine in physiological contexts as well as pathogenic processes. We also introduce a newly developed histaminergic cell‐monitoring transgenic mouse line (*Hdc*‐BAC‐GFP) that serves as a valuable experimental tool to identify the source of histamine and dissect upstream regulatory signals.

## Graphical abstract

Histamine is a bioactive monoamine that is synthesized by the enzymatic activity of histidine decarboxylase (HDC) in various cells. We summarize the current understanding of the regulatory mechanism of *Hdc* gene expression and the roles played by histamine in physiological contexts and pathogenic processes. We also introduce a newly developed histaminergic cell‐monitoring transgenic mouse line (*Hdc*‐BAC‐GFP) that serves as a valuable experimental tool to identify the source of histamine and dissect upstream regulatory signals.

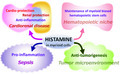

## 1 PATHOPHYSIOLOGICAL ROLES OF HISTAMINE VIA HISTAMINE RECEPTORS

**FIGURE 1:**
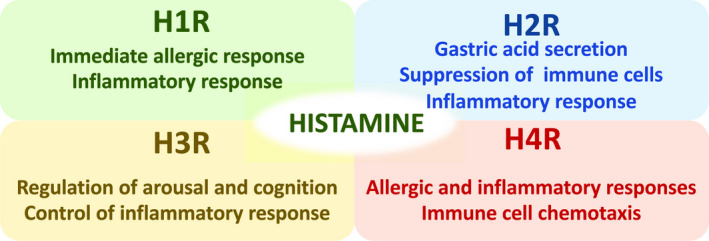
Primary function of histamine through the four types of histamine receptors

Histamine is synthesized through decarboxylation of l‐histidine by the enzymatic activity of HDC and stored in the intracellular granules of basophils and mast cells. When antigen–IgE complex binds to the high‐affinity IgE receptor (FcεRI) on the surface of basophils and mast cells, the stored histamine is promptly released and reaches relatively high local concentration (Gilfillan & Tkaczyk, 2006). Histamine exerts a wide range of pathophysiological activities through four types of specific G protein‐coupled receptors: histamine H1, H2, H3 and H4 receptors (Figure 1; reviewed in Tiligade & Ennis, 2020). Histamine H1 receptor (H1R) is globally expressed in various tissues, including bronchial smooth muscle cells and vascular smooth muscle cells. When histamine binds to H1R, it elicits airway contraction, vascular relaxation, vascular permeabilization and mucosal secretion. Consequently, the type I immediate allergic responses emerge, including bronchial asthma and anaphylaxis.

In the stomach, gastric ECL cells secrete histamine upon stimulation with gastrin and acetylcholine. Thereafter, secreted histamine binds to the histamine H2 receptor (H2R) in parietal cells and activates gastric acid secretion from parietal cells (Tanaka et al., 2002). A series of studies have shown that histamine exerts immunomodulatory activity through H2R signaling (Branco, Yoshikawa, Pietrobon, & Sato, 2018). For instance, histamine represses the release of histamine itself from basophils and mast cells (Bissonnette, 1996; Lichtenstein & Gillespie, 1973; Masini, Blandina, Brunelleschi, & Mannaioni, 1982), represses the proliferation of lymphocytes (Wang & Zweiman, 1978), diminishes neutrophil infiltration (Anderson, Glover, & Rabson, 1977) and suppresses cytokine production from macrophages through H2R (Azuma, Shinohara, Wang, Hidaka, & Ohura, 2001).

The histamine H3 receptor (H3R) functions as an inhibitory autoreceptor at the presynaptic membrane of neurons in the central nervous system and inhibits neuronal release of histamine and other neurotransmitters, including glutamate, γ‐aminobutyric acid (GABA), dopamine, noradrenaline and acetylcholine (Haas, Sergeeva, & Selbach, 2008; Tiligade & Ennis, 2020). In the CNS, histamine regulates arousal and cognition through the activity of H3R. Based on this activity, pitolisant, a potent H3R antagonist, has recently been approved for the treatment of sleep disorders (Kollb‐Sielecka et al., 2017). The function of H3R in the inflammatory response has been described in nervous systems. It was reported that H3R participates in neurogenic control of blood–brain permeability and inflammatory response, and thereby eliminates excessive inflammation in the CNS (Dimitriadou et al., 1994; Krementsov et al., 2013). Another study showed that H3R signaling reduces susceptibility to autoimmune encephalomyelitis in mouse models and attenuates peripheral inflammatory signals (Teuscher et al., 2007).

The histamine H4 receptor (H4R) is mainly expressed in immunocompetent cells, including mast cells, eosinophils, monocytes, dendritic cells and T cells, and H4R promotes immune cell chemotaxis and allergic and inflammatory responses (Walter, Kottke, & Stark, 2011). H4R antagonists have been shown to have anti‐inflammatory and anti‐allergic efficacy in preclinical models of asthma, colitis, dermatitis and arthritis (Cowden et al., 2014).

## 2 MECHANISM OF *Hdc* GENE REGULATION

Transcriptional regulation of *Hdc* gene expression has been analyzed mostly by cultured cell‐based reporter assays, and several different stimuli have been shown to activate *Hdc* promoter activity (summarized in Hirasawa, 2019). Intraperitoneal administration of LPS (lipopolysaccharide) or inflammatory cytokines, including IL‐1 (interleukin‐1) or TNF‐α (tumor necrosis factor‐α), induces the *Hdc* expression in various tissues. A previous report showed that DNA methylation in the promoter sequences of the *Hdc* gene is crucial for transcriptional repression of *Hdc* gene in mast cell lines (Kuramasu, Saito, Suzuki, Watanabe, & Ohtsu, 1998; Suzuki‐Ishigaki et al., 2000). Another study showed that LPS treatment promotes the binding of transcription factor specificity protein 1 (SP1) at a GC box that is located in the promoter region, and thereby enhances *Hdc* gene expression (Hirasawa, Torigoe, Kano, & Ohuchi, 2006). Whether the SP1 binding induces demethylation of the GC box would be of interest. In a gastric cancer cell line, *Hdc* gene expression is activated by gastrin signaling through gastrin‐responsive elements in the promoter region (Ai, Liu, Langlois, & Wang, 2004). This induction mechanism is mediated by eviction of KLF4 (Kruppel‐like factor 4) that functions as a negative regulator of the *Hdc* expression (Ai et al., 2004). KLF4 represses the *Hdc* expression by competing with SP1 in the KLF4/SP1 composite binding site at the promoter GC box. Gastrin treatment diminishes the binding of KLF4 in the GC box, which allows accessibility of SP1 and consequently activates the *Hdc* expression (Ai, Zheng, Yang, Liu, & Wang, 2007). More recently, it was reported that KLF4 suppresses the *Hdc* expression in the bone marrow‐derived mast cells, as well (Nishimura et al., 2020).

## 3 *Hdc*‐BAC‐GFP MOUSE MONITORS HISTAMINE‐PRODUCING CELLS

**FIGURE 2:**
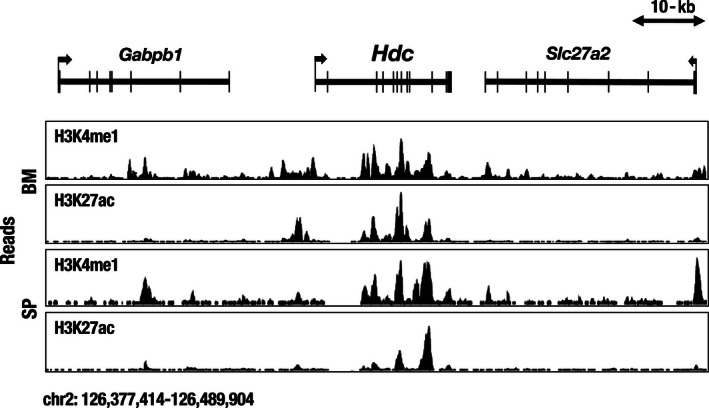
Accumulation of H3K4me1 (histone H3 lysine 4 monomethylation) and H3K27ac (histone H3 lysine 27 acetylation) in the gene body and flanking regions of the mouse *Hdc* locus on chromosome 2 in bone marrow (BM) and spleen (SP) cells. The data were obtained from the UCSC Genome Browser (http://genome.ucsc.edu)

The tissue and cell lineage‐specific regulation of *Hdc* gene expression has rarely been analyzed in vivo. Therefore, we attempted to elucidate this issue by exploiting a transgenic mouse reporter assay system. To this end, we initially tested the in vivo regulatory activity of 1‐kb *Hdc* gene minimal promoter DNA sequences. However, this small fragment failed to recapitulate the endogenous tissue‐specific *Hdc* gene expression profile in the reporter transgenic mouse. We surmised that tissue‐specific *cis*‐regulatory elements for the *Hdc* gene are scattered across a broad range of franking and intron regions of the *Hdc* locus. Mouse and human *Hdc* loci are both composed of twelve exons spanning a 24‐kb genomic region (Figure 2). The accumulation of H3K4me1 (histone H3 lysine 4 monomethylation) and H3K27ac (histone H3 lysine 27 acetylation), a combination of which represent active enhancer elements, is detected throughout the *Hdc* gene body and the 5′‐ and 3′‐flanking sequences in bone marrow and splenic cells, suggesting that multiple *cis*‐regulatory elements participate in *Hdc* gene regulation in various lineages of cells (Figure 2).

**FIGURE 3:**
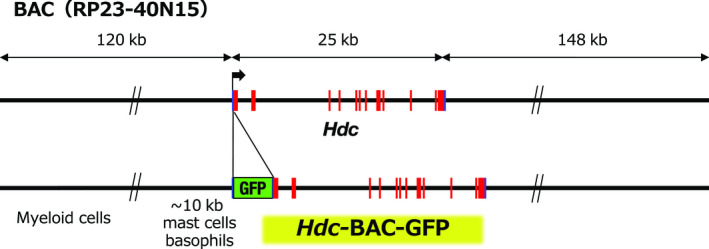
The 293‐kb BAC clone (RP23‐40N15) containing all *Hdc* exons and 120 kb of 5′ and 148 kb of 3′ flanking sequence was modified by inserting GFP (green fluorescence protein) cDNA into the 1st exon. The mast cell‐ and basophil‐specific *cis*‐regulatory elements are located in proximity to the *Hdc* gene. Other myeloid lineage cell‐directed elements are located further than the 10‐kb upstream region

To thoroughly examine the scattered *cis*‐regulatory elements in the *Hdc* locus, we used a 293‐kb bacterial artificial chromosome (BAC) clone encompassing 120‐kb 5′ upstream to 148‐kb 3′ downstream sequences along with 24‐kb *Hdc* structural gene sequences (Figure 3). We constructed a reporter BAC construct by inserting green fluorescence protein (GFP) cDNA by homologous recombination in *Escherichia coli*. Subsequently, we generated two independent lines of BAC transgenic mice (*Hdc*‐BAC‐GFP). Scarce availability of useful antihistamine antibody had been hampering clear detection of histamine‐producing cells in vivo. Therefore, we hoped that the *Hdc*‐BAC‐GFP could monitor the histamine‐producing cells. As anticipated, the *Hdc*‐BAC‐GFP transgenic mice faithfully recapitulated the endogenous *Hdc* gene expression pattern, including peritoneal mast cells, bone marrow basophils, gastric ECL cells and hypothalamic histaminergic neurons (Takai et al., 2019). Subsequent mass spectrometry analysis showed that the GFP‐positive cells contained high level of histamine (Takai et al., 2019). Thus, the *Hdc*‐BAC‐GFP transgenic mice enable specific and quick detection of the histamine‐producing cells by histological as well as flow cytometric analyses.

A recent closer epigenomic analysis showed that H3K4me1 and H3K27ac were accumulated around −8.8‐kb upstream region of the *Hdc* gene in bone marrow‐derived mast cells (Li et al., 2018). The transcriptional factors GATA2 and MITF participate in the activation of *Hdc* gene expression through the −8.8‐kb regulatory elements in the mast cells (Li et al., 2018). Both lines of our *Hdc*‐BAC‐GFP transgenic mice harbored −8.8‐kb upstream sequences, and so, both lines of mice recapitulate mast cell‐specific GFP reporter expression.

Structural configuration analysis of the *Hdc*‐BAC‐GFP transgene in these two independent lines of mice showed that the 1st line harbors 5′ far distal flanking sequences beyond the −10‐kb region, whereas the 2nd line carries a relatively short 5′ regulatory region of <10 kb. We found that the 1st line of *Hdc*‐BAC‐GFP mouse directed the GFP reporter expression in mast cells and bone marrow basophils, as well as other immature myeloid lineage cells (Takai et al., 2019). Meanwhile, the 2nd line of our *Hdc*‐BAC‐GFP mice carrying the short 5′ region showed the GFP reporter expression more specifically in the mast cells and basophils, but not in other immature myeloid lineage cells (Takai et al., 2019). These results suggest that mast cell‐ and basophil‐specific regulatory elements were located in proximity (i.e., <10 kb) 5′ to the promoter region. For other histamine‐producing myeloid lineage cells, putative distal regulatory elements might be required, which are deductively located far beyond the 10‐kb upstream region of the *Hdc* locus (Figure 3).

Yang et al. generated another line of transgenic GFP reporter mice, using BAC DNA containing a similar length of the mouse *Hdc* gene locus. They reported robust GFP reporter expression in CD11b^+^Gr‐1^+^ myeloid lineage cells in the bone marrow, which is consistent with our observations (Yang et al., 2011). However, their reporter mice failed to direct the GFP expression in mast cells in multiple tissues, presumably due to positional effect variegation.

## 4 FUNCTION OF HISTAMINE IN MYELOID LINEAGE CELLS

**FIGURE 4:**
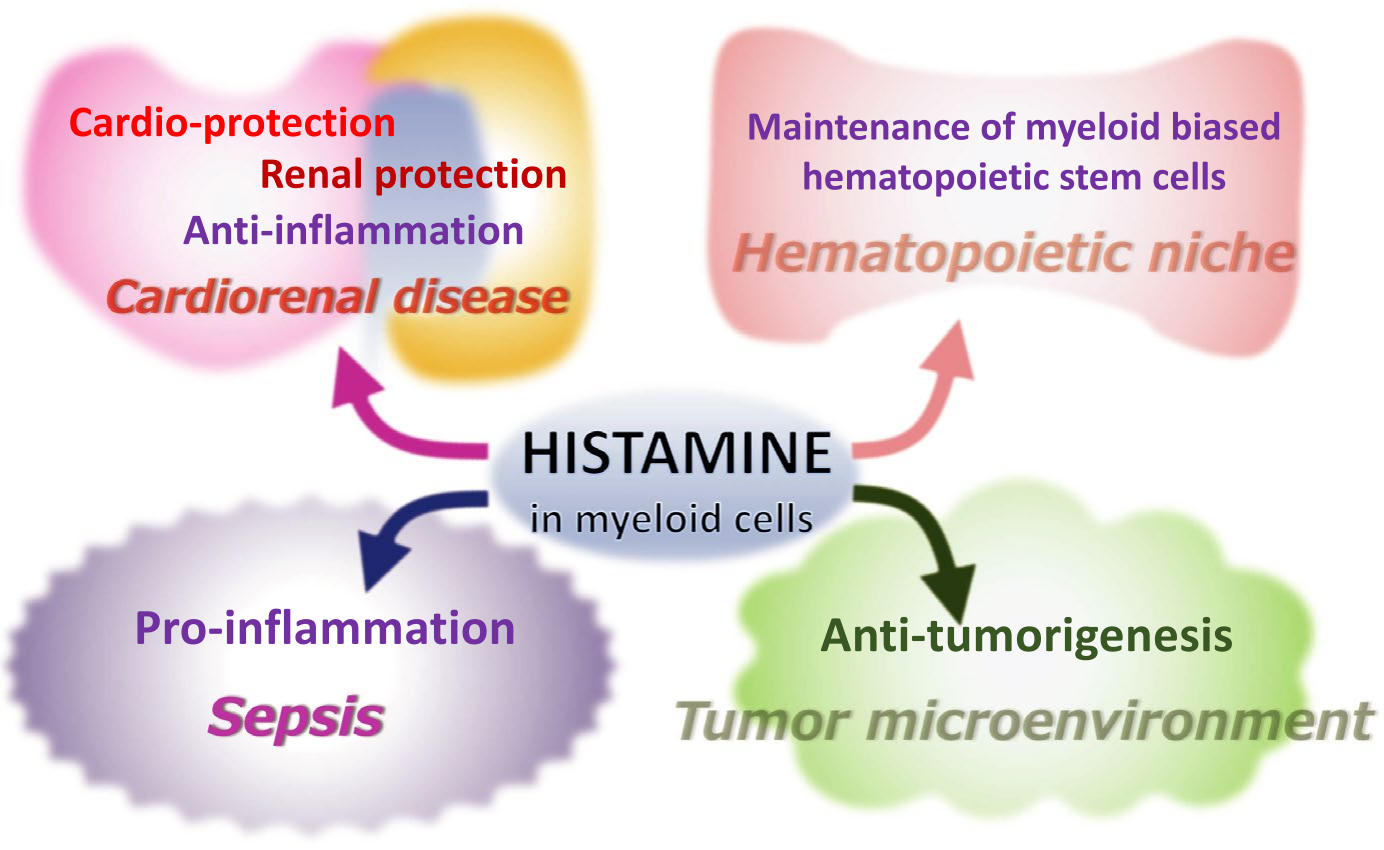
Examples of the pleiotropic functions of histamine produced in myeloid lineage cells

The *Hdc* gene is expressed in various myeloid lineage cells, including dendritic cells, macrophages, neutrophils and immature myeloid progenitors under various pathophysiological conditions (summarized in Hirasawa, 2019). A series of studies show that histamine in these cells participates in the immunomodulation under various disease processes (Figure 4). A recent study showed that the histamine produced by a subset of myeloid lineage cells maintains dormancy of myeloid‐biased hematopoietic stem cells (MB‐HSCs) in the hematopoietic niche through H2R signaling (Figure 4) (Chen, Deng, et al., 2017). MB‐HSCs give rise to myeloid lineage progenitors on demand and supply inflammatory leukocytes upon infection and inflammation. Therefore, histamine may play a role for immune and inflammatory responses by maintaining the MB‐HSCs.

It has been reported that in myocardial infarction (MI) patients, serum histamine levels are significantly increased, and this trend is reproduced in an animal model of MI (Clejan et al., 2002; Zdravkovic et al., 2011). Analysis in the mouse model of MI showed that CD11b^+^ myeloid lineage cells accumulate in the damaged cardiac muscles and are responsible for histamine secretion (Chen, Hong, et al., 2017). The increased histamine inhibits the proliferation of heart fibroblasts via H1R and H2R and thus prevents adverse cardiac fibrosis (Figure 4; Chen, Hong, et al., 2017).

More recently, an interesting study showed that histamine plays an anti‐inflammatory role by suppressing inflammatory cytokine gene expression through the histamine H3 receptor (H3R) in cardiorenal syndrome animal model (Figure 4) (Noguchi et al., 2020). This study showed that an H3R agonist (immethridine) had a preventive efficacy toward chronic heart and kidney diseases (Noguchi et al., 2020). H3R functions as a presynaptic inhibitory autoreceptor and diminishes the release of neurotransmitters (Haas et al., 2008; Tiligade & Ennis, 2020). H3R signaling reportedly eliminates neurogenic activation of the immune response, which might underlie the anti‐inflammatory efficacy of the H3R agonist (Krementsov et al., 2013).

The implication of histamine in cancer biology has been receiving broad interest. It has been generally recognized that tumorigenesis is associated with the recruitment of CD11b^+^Ly6G^+^ tumor‐associated neutrophils (TANs) (Flavell, Sanjabi, Wrzesinski, & Licona‐Limon, 2010; Mantovani, Sozzani, Locati, Allavena, & Sica, 2002). In particular, N2‐type TANs support tumor growth by participating in the tumor‐supportive microenvironment (Fridlender et al., 2009). It has been shown that histamine promotes the cellular differentiation of TANs through H1R or H2R, which diminishes the tumor‐supportive microenvironment; therefore, histamine may function against tumorigenesis (Figure 4) (Yang et al., 2011).

## 5 IMMUNOMODULATORY ROLE OF HISTAMINE IN SEPSIS

Sepsis is defined as systemic inflammatory response syndrome (SIRS), and the mortality can reach 25%–40% (Singer et al., 2016). Despite increasing demand for the sepsis treatments, the mechanistic aspects underlying multiorgan failures in sepsis remain largely elusive. Under septic conditions, pro‐inflammatory cytokines, such as TNFα and IL‐6, evoke cytokine storms, which are responsible for tissue damage during sepsis. Septic patients show increased histamine levels in peripheral blood, suggesting potential involvement of histamine in the pathogenesis of sepsis (Neugebauer et al., 1996). Animal models of sepsis with cecal ligation puncture (CLP) or administration of LPS are associated with increased *Hdc* expression and increased histamine levels in the liver, kidney and lung (Hattori et al., 2016). Analysis using *Hdc*‐deficient mice showed that the increase in TNFα, IL‐1b, IL‐6 and MCP1 levels in sepsis was attenuated when there was a lack of histamine, indicating that the induced histamine promotes the production of the pro‐inflammatory cytokines (Hattori et al., 2016). Many of these pro‐inflammatory cytokines are under the regulation of the transcription factor NF‐κB. In the lungs of *Hdc*‐deficient mice, nuclear levels of NF‐κB under CLP‐induced sepsis were significantly lowered (Hattori et al., 2016). This result suggests that histamine enhances NF‐kB activity and thereby induces the synthesis of pro‐inflammatory cytokines and chemokines. Analysis using H1R and H2R knockout mice showed that this effect was mediated by H1R and H2R (Hattori et al., 2016). In contrast, a separate study reported that the *Hdc*‐deficient mice exhibited enhanced recruitment of macrophages and neutrophils in peritoneal cavity upon bacterial inoculation (Hori et al., 2002). Consequently, the *Hdc*‐deficient mice showed the increased level of serum pro‐inflammatory cytokines, suggesting anti‐inflammatory activity of histamine (Hori et al., 2002). These pleiotropic activities of histamine should be precisely assessed in each disease stage during sepsis progression.

## 6 HISTAMINE‐PRODUCING CELLS IN SEPSIS

Identification of the histamine‐producing cells under septic conditions will be valuable in understanding the etiology and developing an efficient therapeutic strategy. To delve into this, we induced sepsis in the *Hdc*‐BAC‐GFP mouse by LPS administration and examined whether the tissue distribution and the number of histamine‐producing cells were altered. We found that the GFP‐positive histamine‐producing neutrophils were dramatically increased in lung and circulating peripheral blood under septic conditions (Takai et al., 2019). Mass spectrometry analysis showed that the GFP fluorescence level was increased in parallel with the increased histamine synthesis in the neutrophils upon LPS administration (Takai et al., 2019). These results show that the infiltrating neutrophils may be the major histamine source populations in the septic lung and thus can be a hopeful target for treatment (Takai et al., 2019). *Hdc*‐BAC‐GFP mouse will be useful to identify source of histamine in various other disease contexts as well.

## 7 PERSPECTIVE

To achieve a comprehensive understanding of the histamine‐mediated etiology of allergic and inflammatory diseases, elucidation of the histamine‐producing population in each disease condition is undoubtedly of importance. In particular, recent studies shed light on histamine‐producing myeloid lineage cells that are responsible for various pathological processes as well as physiological homeostasis (Figure 4). Further studies are needed to clarify the regulatory mechanism of *Hdc* gene induction in these myeloid lineage cells. For these purposes, the *Hdc*‐BAC‐GFP mouse will serve as a valuable experimental tool to dissect the upstream signals and clarify putative *cis*‐regulatory element(s) that activate *Hdc* gene expression. Additionally, the *Hdc*‐BAC‐GFP mouse would be useful to detect histamine‐producing cell population both in physiological and disease conditions. By identifying these cellular and molecular mechanisms, we can begin to understand the etiological basis and open new therapeutic avenues to circumvent allergic and inflammatory diseases.
